# The Clinicopathological Significance of YAP/TAZ Expression in Hepatocellular Carcinoma with Relation to Hypoxia and Stemness

**DOI:** 10.3389/pore.2021.604600

**Published:** 2021-03-01

**Authors:** Hyunjin Park, Yangkyu Lee, Kiryang Lee, Hyejung Lee, Jeong Eun Yoo, Soomin Ahn, Young Nyun Park, Haeryoung Kim

**Affiliations:** ^1^ Department of Pathology, Seoul National University College of Medicine, Seoul, Korea; ^2^ Department of Pathology, Gangnam Severance Hospital, Yonsei University College of Medicine, Seoul, Korea; ^3^ Department of Pathology, Seoul National University Bundang Hospital, Seongnam, Korea; ^4^ Department of Pathology, Seoul National University Hospital, Seoul, Korea; ^5^ Department of Pathology, Brain Korea 21 PLUS Project for Medical Science, Yonsei University College of Medicine, Seoul, Korea; ^6^ Department of Pathology and Translational Genomics, Samsung Medical Center, Sungkyunkwan University School of Medicine, Seoul, Korea

**Keywords:** hepatocellular carcinoma, Hippo pathway, hypoxia, stemness, immunohistochemistry

## Abstract

**Background/Aims:** Yes-associated protein (YAP) and transcriptional co-activator with PDZ-binding motif (TAZ) activation has been implicated in hepatocarcinogenesis and hepatic progenitor cell differentiation, and hypoxia has been shown to induce nuclear translocation of YAP in cancer cells. Here, we aimed to investigate the relationship between hypoxia, YAP and TAZ expression and stemness-related marker expression in human hepatocellular carcinomas (HCCs) and its clinical implications.

**Methods:** Immunohistochemical stains were performed on tissue microarrays from 305 surgically resected HCCs, and the expression status of YAP and TAZ were correlated with CAIX, stemness markers (K19, EpCAM) and epithelial-mesenchymal transition (EMT)-related markers (uPAR, ezrin). The clinicopathological significance of YAP/TAZ expression was analyzed with relation to CAIX expression status.

**Results:** YAP and TAZ expression were seen in 13.4 and 4.3% of HCCs, respectively. YAP/TAZ-positive HCCs frequently demonstrated higher serum AFP levels, microvascular invasion, advanced tumor stage, increased proliferative activity and expression of stemness- and EMT-related markers, CAIX, p53 and Smad2/3 (*p* < 0.05, all). Interestingly, YAP/TAZ-positivity was associated with microvascular invasion, higher serum AFP levels, stemness and EMT-related marker expression only in tumors expressing CAIX (*p* < 0.05, all), while these associations were not seen in CAIX-negative HCCs.

**Conclusions:** YAP/TAZ expression is associated with vascular invasion, stemness and EMT in HCCs with hypoxia marker expression. The effect of Hippo signaling pathway deregulation in HCC may depend on the presence or absence of a hypoxic microenvironment, and hypoxia marker expression status should be taken into account when considering the use of YAP/TAZ as markers of aggressive biologic behavior in HCC.

## Introduction

Hepatocellular carcinoma (HCC) is the sixth most common malignancy worldwide and the fourth leading cause of cancer-related deaths. A subset of HCCs (5–20%) have been demonstrated to express markers associated with “stemness,” including keratin 19 (K19), epithelial cell adhesion molecule (EpCAM), CD133, CD117/c-kit and SALL4, and these HCCs have been associated with aggressive biological behavior and poor survival [1]. The molecular features of stemness-related marker-expressing HCCs are still not well understood – so far, this group of HCCs has been placed in the G1, S2 and proliferation class molecular subgroups, and *TP53* mutations, transforming growth factor (TGF)-β and Notch signaling pathways have been implicated in these tumors [2–4]. Even though the prognostic relevance of stemness-related marker expression has been increasingly recognized over the recent years, the lack of therapeutic options for this particular subset of HCCs is still a point of concern.

The Hippo pathway controls tissue homeostasis, including organ size, cell proliferation and apoptosis [5, 6]. The oncoproteins Yes-associated protein (YAP) and transcriptional co-activator with PDZ-binding motif (TAZ) are downstream components of this pathway, and deregulation of the Hippo pathway with activation of YAP and TAZ has been implicated in carcinogenesis, including HCC development [7, 8]. For example, *sav1* knockout mice have been shown to develop HCC through the activation of YAP [9]. Activated YAP has been associated with aggressive behavior of various malignant neoplasms, including cell proliferation, stemness, epithelial-mesenchymal transition (EMT) and chemoresistance [10–14]. YAP protein expression has been demonstrated in stemness-marker expressing HCCs and in combined hepatocellular-cholangiocarcinomas, both of which have been postulated to show features of hepatic progenitor cell differentiation [11, 15].

Hypoxia has been associated with aggressive tumor behavior [16]. HCC is no exception; the rapid growth and the relative lack of blood supply results in a hypoxic microenvironment, and HCCs adapt to this unfavorable condition by expressing hypoxia-inducible factors, such as HIF1α and carbonic anhydrase-IX (CAIX) [17, 18]. Associations between CAIX expression, stemness-related marker expression and aggressive clinicopathological features have been seen in HCCs [19]. In addition, hypoxia has been demonstrated to induce YAP nuclear translocation in cancer cells [20]. Therefore, we sought out to investigate the relationship between hypoxia, YAP/TAZ expression and stemness-related marker expression and the clinicopathological implications of YAP/TAZ expression in human HCCs.

## Materials and Methods

### Case Selection

Three hundred and five HCCs that were surgically resected between 2003 and 2012 at Seoul National University Bundang Hospital (SNUBH), Seongnam, Republic of Korea were evaluated in this study. This study was approved by the Institutional Review Board of SNUBH (B-1704-391-301), and informed consent was waived due to the retrospective nature of the study. The clinicopathological information was prospectively recorded by reviewing the electronic medical records, including patient age, sex, underlying etiology, serum alpha-fetoprotein (AFP) levels, preoperative locoregional treatment (including radiofrequency ablation, transarterial chemo/radioembolization), tumor size, gross type [21], multiplicity, histologic grade (Edmonson-Steiner), major vascular and microvascular invasion status, and pathological T and N stages according to American Joint Committee on Cancer (AJCC) 8th edition. Multiplicity was defined as 2 or more tumors, and included intrahepatic metastases and multicentric occurrences. Major vascular invasion was defined as invasion of the main portal vein and first-order branches, the right, middle and left hepatic veins, or the right or left hepatic artery. Microvascular invasion was defined as invasion of smaller caliber vessels by tumor cells; the microvessels were located in the tumor capsule or in the peritumoral non-neoplastic liver, and were not portal vein, hepatic veins or hepatic arteries [22]. The presence of intratumoral fibrous stroma was recorded: fibrous stroma exceeding 10% of the tumor area was regarded as “present.” For the background liver, the fibrosis stage was recorded according to the METAVIR system.

We defined disease-free survival as the interval from surgical treatment to the date of disease recurrence (local recurrence or intrahepatic/distant metastasis). Overall survival was defined as the interval between surgical treatment and date of death from HCC or other causes.

### Tissue Microarray and Immunohistochemistry

Tissue microarray cores of 2 mm-diameter were obtained from the 305 SNUBH HCCs (SuperBioChips Laboratory, Seoul, Korea). One to three cores from the HCCs and matched non-neoplastic liver were evaluated in this study. Immunohistochemical stains for YAP, TAZ, K19, EpCAM, CAIX, p53, ezrin, urokinase-type plasminogen activator receptor (uPAR), smad2/3 and Ki-67 were performed on 4 μm-thick tissue microarray sections manually or by using the Ventana BenchMark GX automated platform (Ventana Medical Systems). The details for each antibody are summarized in Table 1.

**TABLE 1:** List of antibodies used in this study.

Antibody (clone)	Source	Dilution	Method
YAP (rabbit mAb; D8H1X)	Cell Signaling (Danvers, MA, United States)	1:100	Autostainer (CC1, 32′/60′)
TAZ-WWTR1 (rabbit pAb)	Merck (Darmstadt, Germany)	1:100	Autostainer (CC1, 32'/60′)
K19 (mouse mAb)	Dako (Glostrup, Denmark)	1:200	Autostainer (CC1, 32′/60′)
EpCAM (mouse mAb; VU-1D9)	Millipore (Temecula, United States)	1:1,500	Autostainer (CC1, 32'/30′)
CAIX (rabbit pAb)	Abcam (Cambridge, United Kingdom)	1:500	Autostainer (CC1, 32'/60′)
p53 (mouse mAb; DO-7)	Dako (Glostrup, Denmark)	1:1,000	Autostainer (CC1, 32'/16′)
ezrin (mouse mAb; 3C12)	Abcam (Cambridge, United Kingdom)	1:100	Microwave, citrate (pH 6.0)
uPAR (mouse mAb)	Abcam (Cambridge, United Kingdom)	1:40	Microwave, citrate (pH 6.0)
smad2/3 (mouse mAb; C-8)	Santa Cruz (Dallas, United States)	1:500	Autostainer (CC1, 32'/60′)
Ki-67 (mouse mAb; MIB-1)	Dako (Glostrup, Denmark)	1:100	Autostainer (CC1, 64'/16′)

The presence of cytoplasmic expression in >5% of the tumor cells was regarded as positive for K19, uPAR and smad2/3 expression. EpCAM, CAIX and ezrin were expressed in the tumor cell membranes. Although YAP and TAZ were expressed in both the cytoplasm and nuclei, only unequivocal nuclear or nucleocytoplasmic staining for YAP and TAZ were interpreted as positive. Cytoplasmic YAP/TAZ expression without the nuclear stain was not counted as positive regardless of the intensity. Strong nuclear p53 expression in >5% of tumor cells was interpreted as p53 overexpression. The Ki-67 labeling index was calculated as the percentage of Ki-67-stained tumor cell nuclei over the total number of nuclei in a ×400 magnification field, with the use of ImageJ analysis software (downloaded from imagej.nih.gov/ij, version 1.47).

### Statistical Analysis

Statistical analysis was performed using STATA version 14.0 (StataCorp, College Station, TX, United States). The student *t*-test was used for comparing continuous variables, and the chi-square and Fisher-exact tests were used for categorical variables. Survival analysis was performed by the Kaplan-Meier method with the log-rank test. Statistical significance was defined as a *p*-value of < 0.05.

## Results

### YAP/TAZ Expression Status in HCCs and Non-Neoplastic Livers

In non-neoplastic livers, YAP and TAZ were both expressed in the nuclei and cytoplasm of bile ducts and ductular reactions. Hepatocytes were mostly negative for YAP and TAZ; however, occasional periportal or periseptal hepatocytes demonstrated weak nucleocytoplasmic YAP expression. In addition, YAP and TAZ were expressed in endothelial cells. The presence of nuclear stain was regarded as positive for both YAP and TAZ.

**FIGURE 1:**
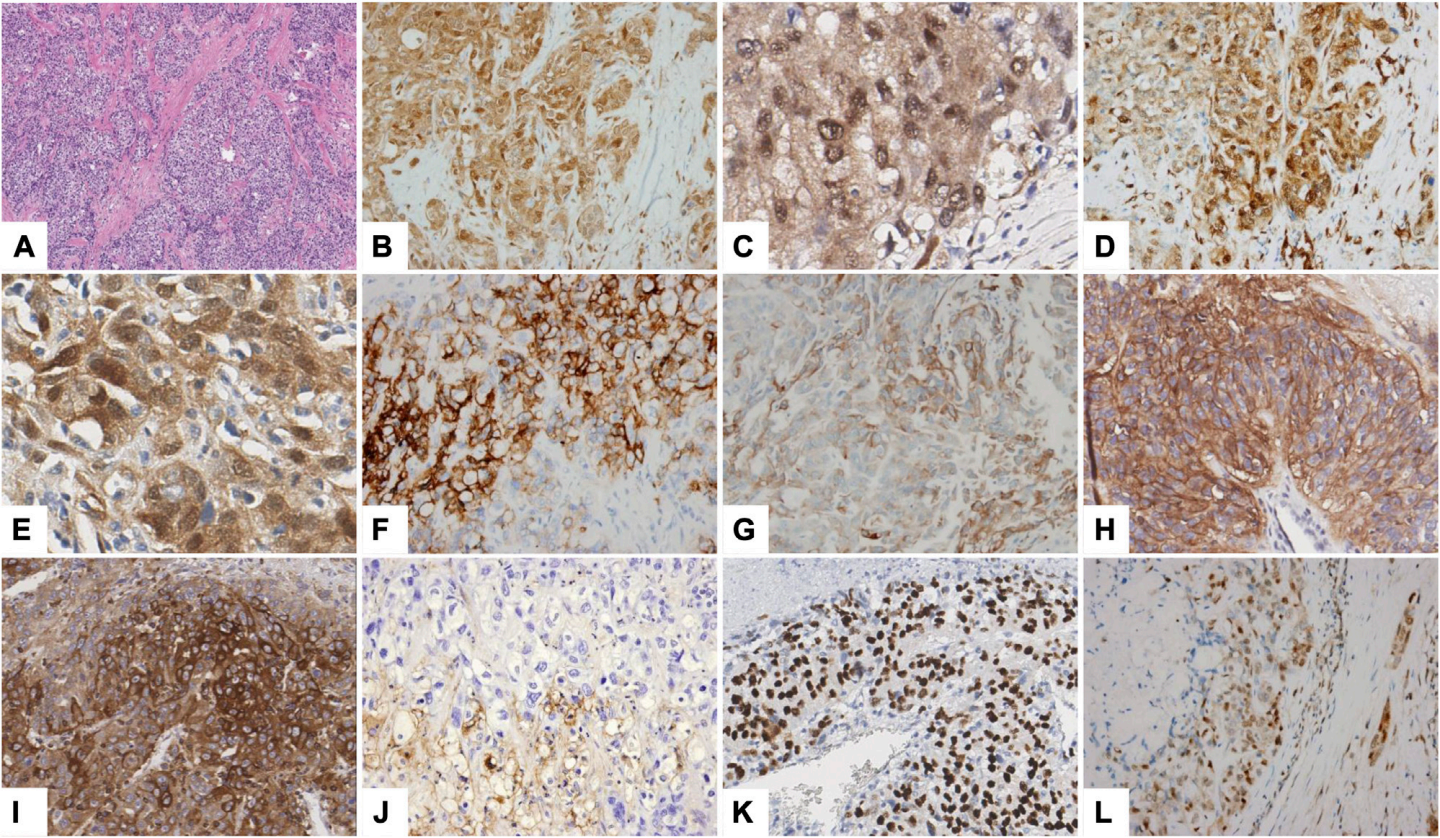
Immunohistochemical stain results. A representative YAP/TAZ-positive HCC showing abundant intratumoral fibrous stroma (**A** hematoxylin-eosin stain, ×100; **B**,**C** YAP; **D**,**E** TAZ) showing expression of CAIX **(F)**, K19 **(G)**, EpCAM **(H)**, ezrin **(I)**, uPAR **(J)**, p53 **(K)** and smad2/3 **(L)** (**B**,**D**,**F**–**L** ×200; **C**,**E** ×400).

YAP and TAZ expression were seen in 41/305 (13.4%) and 13/305 (4.3%) HCCs, respectively (Figure 1). There was a positive correlation between YAP and TAZ expression status (*p* < 0.001) and YAP and TAZ expression was co-localized in the tumor cells for the 13 TAZ-positive HCC cases. There was no significant difference in YAP or TAZ expression frequency according to the HCC etiology. The complete set of immunohistochemical stain data for all ten antibodies was obtained for 255 cases due to tissue core loss, and therefore, 255 cases were enrolled for subsequent analysis. Of the 255 HCCs, YAP and TAZ expression were seen in 34 (13.3%) and 11 (4.3%) cases, and the positive correlation between YAP and TAZ expression was still observed (*p* < 0.001).

### YAP/TAZ Expression is Associated with Aggressive Clinicopathological Features, Hypoxia and Stemness

As a whole, YAP positivity in HCC was associated with significantly more frequent microvascular invasion (*p* = 0.015), a tendency for more frequent portal vein invasion (*p* = 0.059) and significantly more frequent intratumoral fibrous stroma (*p* = 0.035). The proliferation rates were significantly higher in YAP-positive HCCs [mitotic index (*p* = 0.020) and Ki-67 labeling index (*p* < 0.001)]. YAP-positive HCCs demonstrated more frequent K19 (*p* < 0.001) and EpCAM (*p* = 0.001) expression, and more frequent CAIX expression (*p* < 0.001). Serum AFP levels were higher in YAP-positive HCCs (*p* = 0.065), although not statistically significant.

TAZ expression was also associated with more frequent intratumoral fibrous stroma (*p* = 0.046), and significantly increased proliferative activity [mitotic index (*p* = 0.011 and Ki-67 labeling index (*p* < 0.001)]. Similarly to YAP-positive HCCs, TAZ-positive HCCs demonstrated more frequent K19 (*p* = 0.007) and CAIX (*p* = 0.034) expression. EpCAM expression was also more frequent in TAZ-positive HCCs, although not statistically significant (*p* = 0.057).

As there was a significant positive correlation between YAP and TAZ expression status and the clinicopathological features were similar for YAP-positive and TAZ-positive HCCs, we continued our analysis by grouping HCCs expressing YAP and/or TAZ, resulting in 37 (14.5%) YAP/TAZ-positive HCCs and 218 (85.5%) YAP/TAZ-negative HCCs (Table 2). After grouping, YAP/TAZ-positive HCCs were significantly associated with higher serum AFP levels (*p* = 0.024), more frequent microvascular invasion (*p* = 0.011), higher T stage (*p* = 0.017) and lymph node metastasis (*p* = 0.003). YAP/TAZ-positive HCCs were significantly associated with frequent stemness-related marker expression (K19, *p* < 0.001; EpCAM, *p* = 0.002) and frequent CAIX expression (*p* < 0.001). YAP/TAZ-expression was also associated with more frequent expression of EMT-related markers (uPAR: *p* = 0.001, ezrin: *p* = 0.001). In addition, p53 expression was significantly more frequent in YAP/TAZ-positive HCCs (*p* < 0.001), and smad2/3 expression was also increased in YAP/TAZ-positive HCCs (*p* < 0.001), suggesting an association between YAP/TAZ positivity and the TGF-β signaling pathway.

**TABLE 2:** Summary of the clinicopathological and immunohistochemical characteristics of HCCs according to YAP/TAZ and CAIX expression status.

Parameters^a^	Total (n = 255^b^)			CAIX + HCCs (n = 126)			CAIX- HCCs (n = 129)		
	YAP/TAZ+(n = 37)	YAP/TAZ-(n = 218)	*p*-value^c^	YAP/TAZ+(n = 29)	YAP/TAZ-(n = 97)	*p*-value^c^	YAP/TAZ+(n = 8)	YAP/TAZ-(n = 121)	*p*-value^c^
Sex (male/female)	29 (78.4)/8 (21.6)	166 (76.1)/52 (23.9)	0.837	21 (72.4)/8 (27.6)	74 (76.3)/23 (23.7)	0.806	8 (100)/0 (0)	82 (67.8)/29 (24.0)	0.197
Age (years)	56 (30–76)	59 (29–87)	0.260^d^	58 (30–76)	59 (29–83)	0.645^d^	53 (38–72)	59 (30–87)	0.236^d^
Etiology			0.694^e^			0.666^e^			0.799^e^
HBV	29 (78.4)	148 (67.9)		23 (79.3)	68 (70.1)		6 (75.0)	80 (66.1)	
HCV	2 (5.4)	23 (10.6)		1 (3.4)	8 (8.2)		1 (12.5)	15 (12.4)	
HBV + HCV	0 (0)	1 (0.5)		0 (0)	0 (0)		0 (0)	1 (0.8)	
Alcohol	2 (5.4)	10 (4.6)		1 (3.4)	2 (2.1)		1 (12.5)	8 (6.6)	
NAFLD	0 (0)	11 (5.0)		0 (0)	8 (8.2)		0 (0)	3 (2.5)	
Unknown	4 (10.8)	25 (11.5)		4 (13.8)	11 (11.3)		0 (0)	4 (3.3)	
Serum AFP level (≥400 ng/ml)	13 (35.1)	37 (17.0)	0.024	12 (71.4)	22 (22.7)	0.095	1 (12.5)	15 (12.4)	1.000
Preoperative locoregional treatment	12 (32.4)	69 (31.7)	0.849	8 (27.6)	32 (33.0)	0.819	4 (50.0)	37 (30.6)	0.264
Tumor size (cm, median, range)	3.5 (1.5–13.0)	3.3 (0.9–17.0)	0.831^d^	3.5 (1.5–13.0)	4 (0.9–12.5)	0.662^d^	3.1 (1.8–7.0)	3.2 (0.9–17.0)	0.628^d^
Gross type			0.210			0.194			1.000
Vaguely nodular/expanding nodular	12 (32.4)	96 (44.0)		8 (27.6)	42 (43.3)		4 (50.0)	54 (44.6)	
Multinodular confluent/nodular with perinodular extension/infiltrative	25 (67.6)	121 (55.5)		21 (72.4)	55 (56.7)		4 (50.0)	66 (54.5)	
Multiplicity (present)	8 (21.6)	36 (16.5)	0.481	6 (20.7)	18 (18.6)	0.791	2 (25.0)	18 (14.9)	0.609
Edmondson-Steiner grade (III, IV)	28 (75.7)	154 (70.6)	0.694	22 (75.9)	73 (75.3)	1.000	6 (75.0)	81 (66.9)	1.000
Vascular invasion									
Major vascular^f^	5 (13.5)	13 (6.0)	0.154	4 (13.8)	9 (9.3)	0.494	4 (50.0)	43 (35.5)	0.461
Microvascular	23 (62.2)	84 (38.5)	0.011	19 (65.5)	41 (42.3)	0.035	1 (12.5)	4 (3.3)	0.278
Pathologic T category^g^(pT3, pT4)	7 (18.9)	25 (11.5)	0.017	6 (20.7)	15 (15.5)	0.150	1 (12.5)	10 (8.3)	0.566
Pathologic N category^g^(N1) (n = 219)	3 (9.4)	0 (0)	0.003	2 (8.0)	0 (0)	0.056	1 (14.3)	0 (0)	0.061
Intratumoral fibrous stroma (present)	6 (16.2)	14 (6.4)	0.051	8 (27.6)	20 (20.6)	0.451	3 (37.5)	28 (23.1)	0.398
Immunohistochemistry									
CAIX	29 (78.4)	97 (44.5)	<0.001	—	—	—	—	—	—
K19	16 (43.2)	32 (14.7)	<0.001	15 (51.7)	23 (23.7)	0.006	1 (12.5)	9 (7.4)	0.485
EpCAM	20 (54.1)	60 (27.5)	0.002	22 (75.9)	43 (44.3)	0.003	3 (37.5)	40 (33.1)	1.000
uPAR	20 (54.1)	53 (24.3)	0.001	18 (62.1)	31 (32.0)	0.005	2 (25.0)	22 (18.2)	0.642
Ezrin	25 (67.6)	80 (36.7)	0.001	22 (75.9)	48 (49.5)	0.018	3 (37.5)	32 (26.4)	0.682
p53	20 (54.1)	44 (20.2)	<0.001	14 (48.3)	24 (24.7)	0.021	6 (75.0)	20 (16.5)	0.001
Smad2/3	19 (51.4)	15 (6.9)	<0.001	16 (55.2)	12 (12.4)	<0.001	3 (37.5)	3 (2.5)	0.003
Ki-67 labeling index (%)	9.9 (0–44.1)	2.3 (0–50.9)	<0.001^d^	10.2 (0.1–44.1)	3.9 (0–50.9)	0.064^d^	6.9 (0–19.9)	1.3 (0–39.4)	0.094^d^
Mitotic index (/10 high-power fields)	12 (0–116)	5 (0–66)	<0.001^d^	15 (0–70)	8 (0–63)	0.074^d^	7 (1–116)	4 (0–66)	0.024^d^
Background liver fibrosis (n = 252)^h^			0.160^e^			0.639^e^			0.160^e^
F0-F1	36 (16.7)	7 (18.9)		5 (17.2)	18 (18.9)		2 (25.0)	18 (15.0)	
F2-F3	66 (30.7)	8 (21.6)		6 (20.7)	29 (30.5)		2 (25.0)	37 (30.8)	
F4	113 (52.6)	22 (59.5)		18 (62.1)	48 (50.5)		4 (50.0)	65 (54.2)	
Recurrence (%)	23 (62.2)	129 (59.2)	0.715	17 (58.6)	60 (61.9)	1.000	6 (75.0)	69 (57.0)	0.468

^a^ Figures are shown in n (%) for frequency data, and median (range) for continuous variables.

^b^ Only cases with the complete set of immunohistochemical stain data (n = 255) were analyzed.

^c^ Fisher’s exact test, unless otherwise stated (d, e).

^d^ Student t-test.

^e^ Chi-square test.

^f^ Invasion of major branch of hepatic vein or portal vein.

^g^ AJCC TNM staging system, 8th Edition.

^h^ Fibrosis data not available for three cases.

**FIGURE 2:**
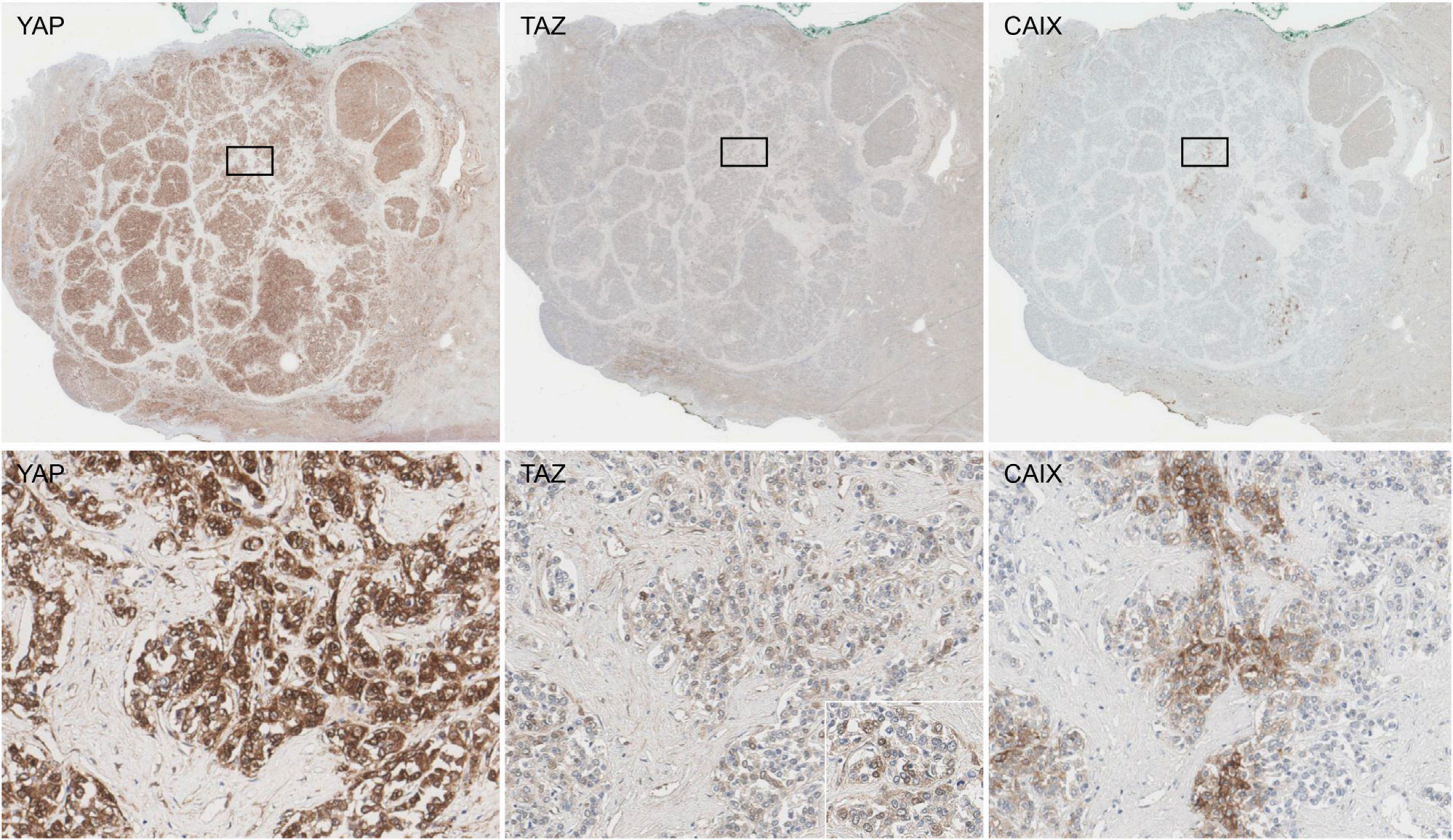
The staining distribution of YAP, TAZ and CAIX in a whole tissue section of a hepatocellular carcinoma **(upper row)**. YAP expression was strong in intensity and easily recognized at low power **(left)**. TAZ expression was focal, but co-localized with YAP expression **(middle)**. CAIX expression was strong in intensity and patchy, and were co-localized with YAP/TAZ expression in most cases **(right)**. The boxed areas in the upper row are demonstrated at ×200 magnification in the lower row. The inset in the lower row is a ×400 magnification picture demonstrating the nuclear staining for TAZ.

To evaluate the distribution of YAP, TAZ and CAIX expression in HCCs in more detail, we selected 10 HCCs from the same tissue microarray cohort that expressed both YAP and TAZ, and stained these tumors for YAP, TAZ and CAIX on whole tissue sections (Figure 2). YAP expression was diffuse homogeneous in 2 cases, while the remaining 8 cases showed patchy expression. TAZ expression was focal in all 10 cases, and frequently localized at the periphery of the tumor cell nests. Although TAZ expression was focal, they were co-localized with YAP; TAZ-positive cells were also YAP-positive in the 10 whole tissue sections tested. CAIX expression was observed in 8 of the 10 YAP/TAZ-positive HCCs, and CAIX staining distribution was patchy in all 8 cases. CAIX expression was co-localized with YAP/TAZ expression in 7 out of the 8 CAIX-positive cases; however, in one case, CAIX expression was not co-localized with YAP or TAZ expression.

### Clinicopathological Significance of YAP/TAZ Expression in HCCs According to Hypoxia Marker Status

**FIGURE 3:**
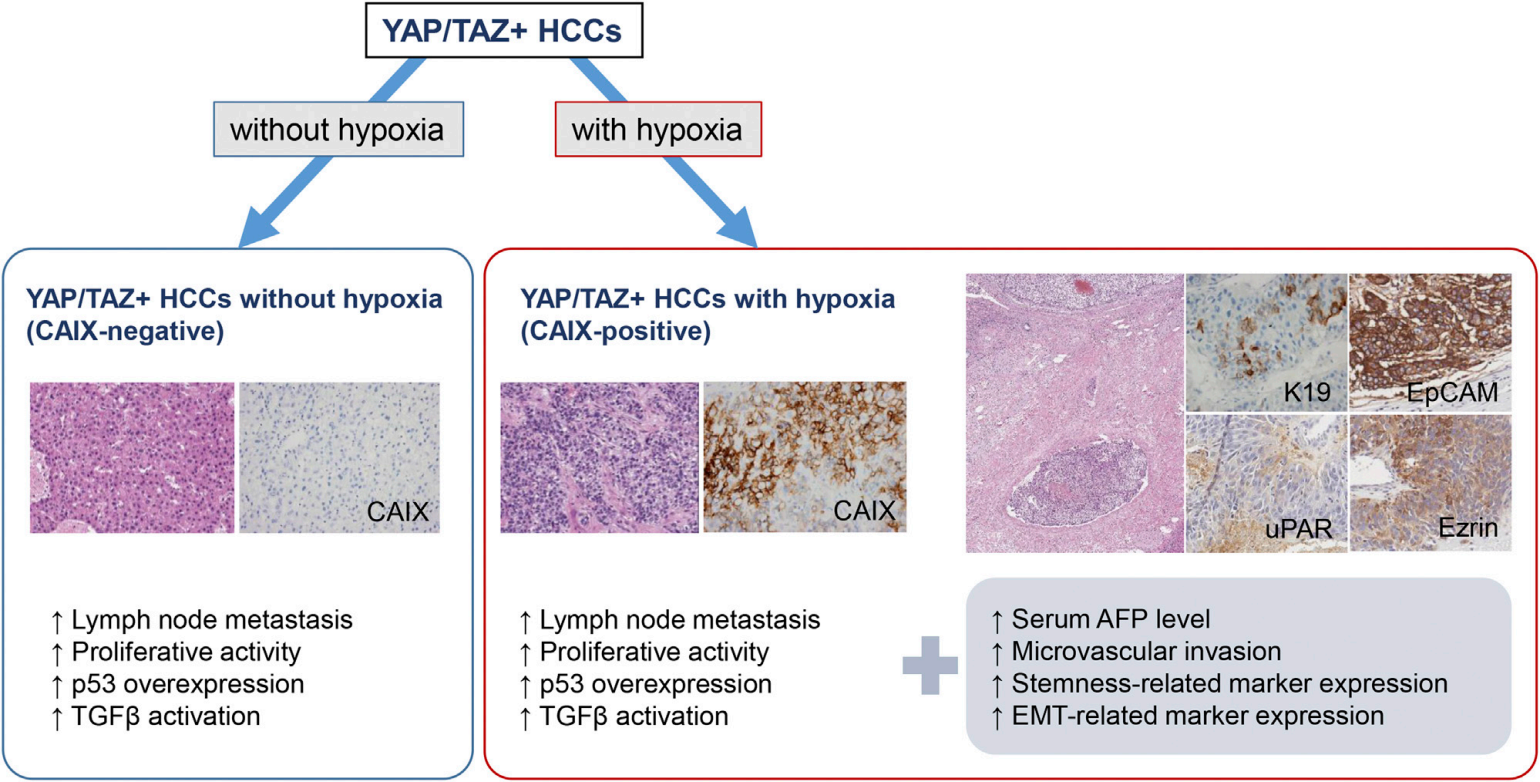
Summary of the characteristics of YAP/TAZ-positive HCCs with relation to hypoxia marker expression status (hematoxylin-eosin stain and CAIX, K19, EpCAM, uPAR and ezrin immunohistochemistry, ×200).

In order to explore the relationship between hypoxia and YAP/TAZ in HCC, CAIX-positive and CAIX-negative HCCs were analyzed separately. The results are summarized in Table 2 and Figure 3. Interestingly, for CAIX-negative HCCs, the expression status of YAP/TAZ had no associations with the expression status of stemness-related or EMT-related markers. On the other hand, for CAIX-positive HCCs, YAP/TAZ expression was significantly associated with K19 (*p* = 0.006), EpCAM (*p* = 0.003), uPAR (*p* = 0.005) and ezrin (*p* = 0.018) expression.

Smad2/3 and p53 expression status was correlated with YAP/TAZ status for both groups, regardless of hypoxia marker status. Similarly, mitotic indices were higher in YAP/TAZ-positive HCCs regardless of CAIX status, although statistically significant only in the CAIX-negative group (*p* = 0.024). Ki-67 labeling indices were higher in YAP/TAZ-positive HCCs regardless of CAIX status, but statistical significance was not reached (CAIX + HCC: *p* = 0.064, CAIX - HCC: *p* = 0.094).

As for the clinicopathological features, more frequent microvascular invasion (*p* = 0.035) was observed in YAP/TAZ-positive HCCs, along with tendencies for higher serum AFP levels (*p* = 0.068) and frequent lymph node metastasis (*p* = 0.056), in the CAIX-positive group. There were no differences in any of the clinicopathological features according to YAP/TAZ expression status in the CAIX-negative group, except for a tendency for more frequent lymph node metastasis (*p* = 0.061). No significant difference in patient survival (overall or disease-free survival) according to YAP or TAZ expression status was demonstrated, regardless of hypoxia status.

## Discussion

In this study, we demonstrate that although the expression of YAP/TAZ in HCCs is associated with “stemness,” EMT and aggressive clinicopathological features as previously reported, this relationship is seen only in HCCs with hypoxia marker expression. The relationship between tumor hypoxia, YAP/TAZ and stemness is still not well understood. Hypoxia is an important condition for the tumor microenvironment, as it can induce cancer growth, metastasis, angiogenesis, EMT and chemoresistance [18]. Wei et al. demonstrated in their *in vitro* and human tissue study of pancreatic cancer that hypoxia deactivated the Hippo pathway, induced the nuclear translocation of YAP and promoted the activation of Snail transcription in the ductal adenocarcinoma cell lines [20]. Interestingly, in an *in vitro* study by Yan et al., hypoxic conditions reduced the expression of the activated form of YAP, while TAZ was induced by hypoxia, suggesting that YAP and TAZ were differentially regulated by hypoxic conditions [23]. In this study, we did not find differences between YAP and TAZ protein expression status when CAIX-positive and CAIX-negative HCCs were analyzed separately. Nevertheless, it is interesting that the expression of YAP and/or TAZ in HCCs was associated with features of aggressive behavior, stemness and EMT only in the presence of CAIX expression, suggesting that the action of YAP/TAZ may be regulated by the hypoxic microenvironment in HCCs.

Another interesting finding in our study is the positive correlation between YAP/TAZ expression, the presence of intratumoral fibrous stroma and the expression of stemness-related markers. YAP expression has previously been demonstrated in a substantial proportion of K19+/EpCAM + HCCs and combined hepatocellular-cholangiocarcinomas [11, 15], and it was therefore suggested that the Hippo signaling pathway may be involved in the acquisition of stemness features of HCCs and combined hepatocellular-cholangiocarcinomas [15]. Notably, TGFβ has been recently shown to interact with Hippo signaling pathways to stimulate regenerating hepatocytes to undergo an EMT-like response [24]. In addition, TGFβ signaling pathway upregulation has been demonstrated in HCCs with abundant fibrous stroma, such as scirrhous HCC; these tumors are frequently associated with stemness-related marker expression [25]. We observed a positive correlation between YAP/TAZ and smad2/3 expression in both CAIX-positive and CAIX-negative HCCs, suggesting that, regardless of hypoxia status, TGFβ signaling may play an important role in the fibrogenesis of HCCs by interacting with the Hippo signaling pathway.

Despite the associations with clinicopathological features of aggressive behavior, the presence of YAP/TAZ expression did not appear to have prognostic significance in our study, even on separately analyzing the CAIX-positive and negative HCCs. There have been discrepancies regarding the prognostic value of YAP and TAZ in the literature, and differences in the interpretation methods (nuclear, cytoplasmic or nucleocytoplasmic) for YAP and TAZ expression has been suggested to be a reason for this discrepancy [11, 14, 26–28]. We interpreted only nuclear staining as positive for YAP and TAZ as reported by Van Haele et al. [11].

From the therapeutic point of view, several small molecule inhibitors targeting the Hippo signaling pathway have been found to be effective in treating various malignancies. For example, dasatinib, which inhibits the nuclear localization of the YAP/TAZ complex, and verteporfin, which disrupts the formation of the YAP-TEAD complex, are currently used in clinical trials for chronic myeloid leukemia and metastatic breast cancer, respectively [29]. Verteporfin has also been demonstrated to suppress the early stages of hepatocarcinogenesis [30]. As we found that the biological behavior of YAP/TAZ-expressing HCCs may depend on the expression status of CAIX, we could carefully suggest that CAIX could potentially be used as a marker of response to drugs targeting the Hippo pathway in HCCs. However, this would need validation with functional studies and also large scale tissue-based studies in independent patient cohorts.

In conclusion, YAP and TAZ expression in HCC are associated with aggressive clinicopathological features, stemness and EMT, and this relationship is seen in HCCs with hypoxia marker expression. This suggests that the effect of Hippo signaling pathway deregulation in HCC may depend on the presence or absence of a hypoxic microenvironment, and that hypoxia marker expression status should be taken into account when considering the use of YAP/TAZ as markers of aggressive biologic behavior in HCC.

## Data Availability

The raw data supporting the conclusions of this article will be made available by the authors, without undue reservation.

## Abbreviations

HBV, hepatitis B virus; HCV, hepatitis C virus; NAFLD, non-alcoholic fatty liver disease; AFP, alpha-fetoprotein; CAIX, carbonic anhydrase-IX; K19, keratin 19; EpCAM, epithelial cell adhesion molecule; uPAR, urokinase-type plasminogen activator receptor.
